# Open issues in Mucopolysaccharidosis type I-Hurler

**DOI:** 10.1186/s13023-017-0662-9

**Published:** 2017-06-15

**Authors:** Rossella Parini, Federica Deodato, Maja Di Rocco, Edoardo Lanino, Franco Locatelli, Chiara Messina, Attilio Rovelli, Maurizio Scarpa

**Affiliations:** 1 UOS Malattie Metaboliche Rare, Clinica Pediatrica dell’Università Milano Bicocca, Fondazione MBBM, ASST Monza e Brianza, Monza, Italy; 2 Division of Metabolic Disease, Bambino Gesù Children’s Hospital, IRCCS, Rome, Italy; 3 Unit of Rare Diseases, Department of Pediatrics, IRCCS “Giannina Gaslini” Children’s Hospital, Genoa, Italy; 4 UOSD Centro Trapianto di Midollo Osseo, Dipartimento Ematologia-Oncologia Pediatrica, IRCCS “Giannina Gaslini” Children’s Hospital, Genoa, Italy; 5 Department of Pediatric Hematology and Oncology, Bambino Gesù Children’s Hospital, IRCCS, Rome, Italy; 6 University of Pavia, Pavia, Italy; 7 Dipartimento di Pediatria, DAI di Salute della Donna e del Bambino, Azienda Ospedaliera-Università di Padova, Padova, Italy; 8 Centro Trapianto di Midollo Osseo, Clinica Pediatrica dell’Università di Milano-Bicocca, Fondazione MBBM, ASST Monza e Brianza, Monza, Italy; 9 Department for the Woman and Child Health, University of Padova, Padova, Italy

**Keywords:** Mucopolysaccharidosis I, Hurler, Allogeneic hematopoietic stem cell transplantation, Enzyme replacement therapy, Metabolic disorder, Lysosomal storage

## Abstract

Mucopolysaccharidosis I-Hurler (MPS I-H) is the most severe form of a metabolic genetic disease caused by mutations of *IDUA* gene encoding the lysosomal α-L-iduronidase enzyme. MPS I-H is a rare, life-threatening disease, evolving in multisystem morbidity including progressive neurological disease, upper airway obstruction, skeletal deformity and cardiomyopathy. Allogeneic hematopoietic stem cell transplantation (HSCT) is currently the gold standard for the treatment of MPS I-H in patients diagnosed and treated before 2–2.5 years of age, having a high rate of success. Beyond the child’s age, other factors influence the probability of treatment success, including the selection of patients, of graft source and the donor type employed. Enzyme replacement therapy (ERT) with human recombinant laronidase has also been demonstrated to be effective in ameliorating the clinical conditions of pre-transplant MPS I-H patients and in improving HSCT outcome, by peri-transplant co-administration. Nevertheless the long-term clinical outcome even after successful HSCT varies considerably, with a persisting residual disease burden. Other strategies must then be considered to improve the outcome of these patients: one is to pursue early pre-symptomatic diagnosis through newborn screening and another one is the identification of novel treatments. In this perspective, even though newborn screening can be envisaged as a future attractive perspective, presently the best path to be pursued embraces an improved awareness of signs and symptoms of the disorder by primary care providers and pediatricians, in order for the patients’ timely referral to a qualified reference center. Furthermore, sensitive new biochemical markers must be identified to better define the clinical severity of the disease at birth, to support clinical judgement during the follow-up and to compare the effects of the different therapies. A prolonged neuropsychological follow-up of post-transplant cognitive development of children and residual disease burden is needed. In this perspective, the reference center must guarantee a multidisciplinary follow-up with an expert team. Diagnostic and interventional protocols of reference centers should be standardized whenever possible to allow comparison of clinical data and evaluation of results. This review will focus on all these critical issues related to the management of MPS I-H.

## Background

Mucopolysaccharidosis type I (MPS I) is a severe, genetic, multisystem disorder caused by a deficiency of the lysosomal enzyme α-L-iduronidase (IDUA), which is responsible for the hydrolysis of glycosidic bonds in terminal α-L-iduronic acid residues of the complex glycosaminoglycans dermatan sulfate and heparan sulfate. Mutations in *IDUA* impair the degradation of these molecules that accumulate within lysosomes triggering a complex cascade of intracellular events ultimately leading to tissue damage and organ dysfunction [1]. MPS I evolves into multisystem morbidity, characterized by relevant clinical variability [1]. Most known cases fall within the severe form (Hurler syndrome), with signs/symptoms starting in the first year of life. They include upper airway obstruction due to mucosal and adenotonsillar hypertrophy and repeated infections, laryngeal and tracheal narrowing, hearing and visual deficit, gargoyle facies, organomegaly, abdominal herniae, valve disease and cardiomyopathy, skeletal deformities such as thoracolumbar gibbus and joint stiffness, and progressive neurological disease with severe cognitive delay. In the absence of specific treatment death occurs typically within the first decade of life. Milder forms of MPS I are also known, with a continuum of different severity phenotypes which is classically divided in two additional phenotypes, attenuated (Scheie syndrome) and intermediate (Hurler-Scheie syndrome), which, in addition to Hurler syndrome, cover the entire spectrum of the disease. Patients with so-called “milder” phenotypes can reach adulthood, but may experience significant morbidity [2].

The currently available treatments for MPS I include allogeneic hematopoietic stem cell transplantation (HSCT) and enzyme replacement therapy (ERT).

Following transplantation of hematopoietic cells collected from donor bone marrow, which was initially proposed for the treatment of MPS I-Hurler (MPS I-H) [3], HSCT has become the gold standard for the treatment of the severe form of MPS I in patients diagnosed and treated before 2–2.5 years of age who have a developmental quotient (DQ) >70 at the time of HSCT [2, 4–6]. Recently, due to the improved safety of this procedure, the therapeutic indication for HSCT has been extended to treat patients with severe Hurler-Scheie who are at risk of progressive neurocognitive impairment [6, 7]. HSCT is the only treatment for MPS I which is successful in halting the progression of cognitive delay; it also acts on other organs/systems in the body slowing progression of damage due to GAGs deposition [2, 6].

Despite the success of this approach, transplanted patients may nonetheless develop a significant burden of disease, especially heart valve disease and osteo-articular complications requiring multiple surgical interventions, and ocular involvement. Conversely, the severity of post-transplant cognitive impairment is usually related to age and/or psychomotor development at the time of transplantation and to completeness of engraftment [8, 9].

ERT with human recombinant laronidase (a polymorphic form of human α-L-iduronidase) is the most diffuse treatment for attenuated MPS I [10, 11] and its safety and efficacy have been proven over the years [12]. It is not recommended for the severe Hurler form because the enzyme is unable to cross the blood-brain barrier. It also does not completely correct bone, heart valvulopathy, and corneal clouding [13], and there are data suggesting better metabolic correction after HSCT compared with ERT [14]. As the overall risk of HSCT is progressively reducing, and considering the burden of weekly 4 h infusions and risk of antibody development and drug related adverse reactions, some authors consider it appropriate to also offer HSCT to patients with the attenuated form [15].

However, there is evidence that the combination of ERT and HSCT neither negatively affects engraftment nor promotes the development of graft-versus-host disease (GVHD) [16], and a recent 10-year follow-up suggests a beneficial effect of peri-transplant ERT [17]. Following this body of evidence, peri-transplant ERT from diagnosis to engraftment is now considered an established indication to relieve somatic disease. Moreover, other uses of ERT in transplanted patients have been suggested, including intrathecal ERT (https://clinicaltrials.gov Identifier NCT00638547), whose efficacy needs to be verified.

This review examines current overall management, therapy and follow-up of MPS I-H, and focuses on unresolved issues that need further discussion and better definition.

### Current management of HSCT in MPS I-H

Since the first use of HSCT in Hurler patients [3], over 500 individuals have received HSCT procedures in more than 30 years [8]. HSCT has been indicated as the first-line therapeutic option for MPS I-H, particularly in children with DQ >70 who are <2 years of age at time of transplantation [2, 18, 19]. The most relevant benefit of HSCT performed in very young patients is that on intellectual impairment, allowing the establishment of intellectual development and functions that otherwise would be lost in non-transplanted patients [2]. The favorable clinical outcome of HSCT hinges mainly upon the age at time of transplantation, which usually, albeit not always, correlates with the DQ of the transplanted child.

It is well established that the sooner HSCT is performed, the better the chances are of a positive outcome [8, 9, 19]. A recent international, multicenter, retrospective analysis on transplantation-related predictors of long-term outcome in 217 MPS I-H patients after HSCT, revealed that performing HSCT at a very early age (<12 months) in patients with normal or mildly impaired cognitive development at baseline offers the best chance for a long-term, favorable cognitive prognosis [8]. However, this retrospective study examined a heterogeneous population of patients, some of whom were transplanted more than 20 years earlier, when transplantation approaches were very different. Poe et al. showed a sharp correlation between age of transplantation and better long-term developmental outcome in 31 transplanted children with Hurler syndrome, an age at transplantation <9 months being associated with improved cognitive outcomes [9]. Early diagnosis is therefore crucial for the optimal outcome of these patients.

Other predictors of better outcome after HSCT include using regimens designed to achieve full-donor chimaerism, the use of non-carrier donors and the choice of graft source [7, 8]. A retrospective study of the European group for Blood and Marrow transplantation (EBMT) showed that a fully myeloablative busulfan-based conditioning regimen with busulfan dose-adjusted according to pharmacokinetics data protected against graft failure [5]. Busulfan has been shown to be instrumental in inducing microglia reconstitution by donor cells [20]. Using non-carrier donors has been shown to be associated with higher enzymatic levels after the allograft, resulting in a more favorable patient outcome in the long-term, including neurocognitive development [8]. Current results in expert centers show high overall survival rate (>90%) with low toxicity and high rate of full donor chimaerism (>90%) [7]. Full-donor chimaerism rate was found to be significantly higher in recipients successfully transplanted with umbilical cord blood compared with receiving either bone marrow or peripheral blood stem cells [7, 10].

Hence, in comparison to a decade ago when the use of HSCT was limited by a high risk of graft failure and transplantation-related morbidity and mortality, HSCT has become much safer [7].

A European consensus procedure, which was established by convening a panel of MPS I experts, adopted a modified Delphi method in order to examine critical issues related to allogeneic HSCT and ERT therapeutic choices for MPS I. The goal was to update clinical guidelines on the optimal management of this debilitating and severe disorder in the presence of uncertainty among healthcare providers [6]. Consensus was reached on the assumption that strategies for optimal management of MPS I should be tailored to the distinct features of individual cases, including age, disease severity, degree and type of clinical involvement, taking in consideration the wide heterogeneity of the clinical course of MPS I patients [2].

National guidelines, such as those issued by the UK National Specialist Commissioning Advisory Group (NSCAG) [21], and by the Italian Society for the Study of Inborn Metabolic Diseases and Newborn Screening (SIMMESN) and the Italian Association of Paediatric Haematology and Oncology (AIEOP), shared similar recommendations [22].

### Open issues of HSCT and other therapies for MPS I-H

As discussed previously, the therapeutic approaches for MPS I have been well established over years of clinical experience. However, unresolved and open issues regarding HSCT have emerged from the clinical experience, including: i). the need of early transplantation for an improved cognitive outcome; ii). the feasibility of granting Hurler-Scheie patients with slowly-evolving progressive mental retardation access to transplantation; iii). the residual burden of osteo-articular complications.

Early intervention in case of severe MPS I is of paramount importance. The treatment algorithm proposed by current guidelines of the International Consensus Panel on the Management and Treatment of MPS I [2] recommends evaluation of the risk/benefit ratio of HSCT for each individual patient according to age, disease phenotype, DQ, severity of disease and potential for growth. Two major thresholds are considered, age and DQ of the patient, with the aim of preserving cognitive abilities. Therefore, HSCT is usually recommended in a patient <2–2.5 years of age and with a DQ >70 if deterioration is anticipated based on clinical evidence (i.e. results of developmental tests or genotype information). On the other hand, in patients <2–2.5 years of age but with a DQ <70, ERT is suggested since a low DQ increases the risk/benefit ratio and reduces the cognitive benefit of HSCT. In contrast, Poe et al. state that age is the most important variable to be considered for HSCT [9]. In this paper, greater gains in cognitive development post-transplantation were associated with younger age (i.e. in patients aged 2 to 8 months old at transplantation) despite the fact that some of these patients had a DQ <70 at baseline. Notably, this study found evidence that HSCT was associated with neurodevelopmental benefits even in patients who were older than 2 years of age at the time of treatment.

The decision to perform HSCT in an infant over 2–2.5 years and <70 DQ at the time of transplantation could be taken individually after discussing with the family, explaining the natural history of the disease with fast progressing severe developmental delay and death around 10 years of age, the risk of the procedure, and the possible expected outcomes being borderline to severe mental delay.

Current recommendations also suggest that HSCT may be considered in patients >2.5 years of age in rare cases, namely in Hurler-Scheie patients with a slightly milder phenotype and DQ ≥70 who are at risk of mental delay [4, 6]. Importantly, the differentiation of hematopoietic stem cells (HSCs) into glial cells allows delivery of the deficient enzyme into the central nervous system, as shown in experimental models [20, 23, 24]. Therefore, HSCT is uniquely able to prevent or delay the impairment of cognitive functions in young children.

Although HSCT can transform the natural history of the disease, the results of long-term studies revealed that, even in the presence of complete donor engraftment and normal enzymatic activity, an enduring osteo-articular residual burden of disease may persist. This includes dysostosis multiplex and scoliosis, which frequently require aggressive surgical intervention and may negatively impact quality of life.

Given that HSCT does not seem to prevent residual osteoarticular deformities, successful HSCT was nonetheless shown to have an early and positive effect on dens morphology in a retrospective study which investigated the effect of HSCT on the craniocervical junction by analyzing sequential magnetic resonance imaging (MRI) in 30 MPS I-H patients (age of transplant 7–23 months, mean 13.5 months) [25]. On the other hand, a recent evaluation of radiographic parameters in 52 patients with MPS I-H demonstrated progressive hip dysplasia over time, despite successful HSCT being performed at a median age of 12 months (range 3–30 months) [26].

Other therapies for MPS I-H are under investigation. Transplantation of genetically manipulated autologous stem cells (ex-vivo gene therapy) following conditioning with busulfan resulted in supra-normal levels of enzyme in metachromatic leukodystrophy, an inherited lysosomal storage disease caused by arylsulfatase A deficiency [27]. Whether supra-normal levels of IDUA in MPS I patients will improve the endpoints described will be tested in experimental gene therapy protocols, which are currently under development [28].

Compared with HSCT, the use of genetically corrected hematopoietic stem cells could have some advantages: decreased risks related to allogeneic HSCT, mainly rejection and graft versus host disease; and availability for patients lacking an HLA- matched donor. Studies of gene therapy in animals have yielded promising results showing detectable expression of IDUA and clearance of pathological GAGs in the brain, as well as improved craniofacial appearance and neurobehavior [29–31]. The effect is probably more striking if the animals are treated at birth as a robust immune system response toward the transgenic protein is less likely than in adulthood. In addition, gene therapy at an early age might halt the well-known complex cascade of cell metabolism modifications which cannot be reverted in animals with established disease [32].

### Need for early intervention and neonatal screening

The observation that hip dysplasia and other bone abnormalities in MPS I-H cannot be corrected by HSCT indicates that bone damage occurs very early in these patients and suggests that pre-symptomatic HSCT might be beneficial. A recent murine model of MPS I, engineered by disruption of *IDUA*, revealed that neonatal bone marrow transplant significantly reduced signs and symptoms of the disease before their appearance [33], thus supporting the indication of pre-symptomatic HSCT. As mentioned previously, transplanting before 9 months of age had an advantage in terms of normal cognitive development over the long-term follow-up period [9]; this observation supports the rationale for newborn screening as patients with MPS I Hurler are often not clinically diagnosed early enough to perform HSCT before 9 months of age.

Several methods for newborn screening of MPS I have been developed to date and a conclusive diagnosis of MPS I may be reached by measuring IDUA activity in rehydrated dried blood spots using high sensitivity, electrospray ionization tandem mass spectrometry (ESI-MS/MS) [34]. Pilot newborn screening programs for MPS based on ESI-MS/MS enzymatic assay, are currently ongoing globally, including a number of regions in Italy (Tuscany, Umbria, Veneto).

Following the detection of enzyme deficiency in a patient, *IDUA* mutations are then investigated in its genome. Notably, according to the first results of a pilot screening program in the US, pseudodeficiency of the enzyme occurs more frequently than expected [35] and must be carefully addressed, considering that a message of probable disease is a devastating communication when reported to the family.

Many studies have contributed to the attempt of correlating mutations with disease severity, but complete results have not been obtained [2, 36]. A wide international *IDUA* mutational profiling revealed the existence of relevant allelic heterogeneity among different countries [37]. This analysis in patients from many European countries showed that genotype was completely informative on phenotype in less than 50% of cases [37].

Presymptomatic newborn patients with *IDUA* deficiency and known severe gene mutations (e.g. nonsense common W402X and Q70X, missense A327P and G51D, and others) identified on both alleles (homozygous or combined heterozygous) are destined to develop severe MPS I due to the absence of any functional enzyme [37, 38] and should be referred for transplantation without delay. Patients with known mild mutations such as R89W and L492P should be considered for treatment with ERT instead, whose start will be decided on a clinical and possibly biochemical basis. The challenging issue will be in the case of new mutations and of those mutations whose phenotypic effect has not yet been clarified. The therapeutic options may be either administering ERT only or initiating ERT and searching for a suitable HSCT donor. Notably, if ERT is administered early to an unclassified patient, the identification of his/her phenotype may be confounded by the early ERT itself. This may prevent or reduce the development of those early somatic changes regarded as hints for the clinical differentiation of mild cases from severe cases. To improve phenotype prediction, an algorithm was proposed to detect severe MPS I patients on the basis of clinical and biochemical data in the first month of life [39]. Moreover, an improved assay of IDUA activity in fibroblasts from skin biopsies of newborn MPS I patients allowed the accurate quantification of residual enzymatic activity that may be useful to predict MPS I phenotype severity [40]. However, these innovative and promising approaches to differentiating patients through the measure of residual enzymatic activity are feasible tests only if performed in a research laboratory with well-recognized expertise in the field. Therefore, further and more sensitive markers of the disease are still needed to allow the identification of case severity of MPS I in the first month of life.

### Peri-transplant ERT

For patients with severe MPS I, peri-transplant administration of laronidase (recombinant IDUA) should be considered as a supportive/adjuvant treatment in addition to the urgency of initiating treatment with HSCT. The long-term safety and effectiveness of replacement with laronidase has been confirmed for non-neurological manifestations in a wide range of MPS I patients of different ages and disease severity [41]. A European consensus panel treatment algorithm has highlighted the beneficial effects of ERT in pre-transplant patients, in terms of respiratory and cardiovascular functions, supporting the inclusion of ERT in treatment protocols of patients assigned to and waiting for allogeneic HSCT [6]. Notably, ERT was reported as being very effective in reverting cardiomyopathy and severe heart failure during a period of a few months in a number of Hurler patients who later underwent HSCT [42, 43]. Furthermore, HSCT attenuated the formation of neutralizing allo-antibodies developing upon ERT [44], reinforcing the strength of a strategy based on concurrent ERT and HSCT in the same treatment protocol [17].

Co-administration of ERT and HSCT was shown to be well tolerated with no increased risk of immune-mediated complications, namely GVHD or graft rejection [45]. ERT may also significantly improve pre-transplant clinical conditions despite its inability to cross the blood-brain barrier and therefore absent/limited beneficial effect on cognitive functions [6, 45].

A recent international study of laronidase-based ERT in patients with MPS I who underwent HSCT established that, beyond age at HSCT, normal IDUA levels attained post-transplant and improvement of pre-transplant clinical conditions were positive prognostic factors for clinical outcome in most organ systems, but not for neurodevelopmental outcome [8]. On the other hand, a 2-year study in 19 children showed that ERT in association with HSCT may contribute to ameliorate the cognitive outcome after transplant [46]. Because intravenous IDUA is not anticipated to cross the blood-brain barrier, this neurodevelopmental improvement may be explained as an indirect effect of a better control of somatic manifestations of the disease by ERT [47].

Based on the evidence of the benefit of ERT in MPS I patients, and in consideration of the progressive nature of the disease, enzyme replacement should be initiated as soon as possible [13] and is generally agreed to start ERT at diagnosis [17, 45]. Notably, the European consensus panel agreed that ERT with laronidase should be initiated in symptomatic patients with MPS I at the time of diagnosis and also prior to HSCT, which must not be delayed, and that ERT was not associated with a reduced engraftment rate [6].

A number of ongoing clinical studies are examining the effect of prolonged treatment with laronidase (https://clinicaltrials.gov Identifiers NCT01173016, and NCT01572636), and intrathecal administration (https://clinicaltrials.gov Identifier NCT00638547) associated with HSCT. Once completed, it is hoped these studies will provide interesting insight on long-term treatments with ERT.

Currently, most transplant centers are administering ERT to MPS I-H patients prior to HSCT and continuing it until achievement of donor-derived engraftment. In case of graft failure, ERT is usually resumed until a second transplant is performed and engrafted. Chimaerism and/or leukocyte IDUA enzymatic activity may be suitable markers to decide whether or not ERT has to be resumed after HSCT [8]. ERT in combination with HSCT seems safe with no deleterious effects on engraftment. However, whether ERT should be continued for a longer time period after a successfully engrafted first HSCT (i.e. 6–12 months after HSCT when donor cells should have largely replaced tissue macrophages and microglia) needs to be addressed by further specific studies.

### Post-transplant ERT

A significant residual disease burden may persist after HSCT involving non-progressive mental retardation, orthopedic manifestations, and damage to various organs including, mitral and aortic value deformities, decreased visual acuity and chronic ear infections [4]. Consequently, ERT treatment has been performed in an attempt to counteract the somatic disease manifestations experienced by most patients after successful HSCT, in some cases several years beyond the peri-transplant period.

The only published case report describes a male patient who underwent a successful HSCT procedure at 2.5 years of age and who presented with progressive respiratory failure at the age of 14 years, despite good donor chimaerism and 50% of normal IDUA activity, matching that of his donor sibling. The patient was treated with weekly laronidase accompanied by non-invasive ventilation for 24 month. Within the therapy period his respiratory functions significantly improved, as did his quality of life [48].

Other few cases of positive impact of ERT administered a number of years after successful HSCT have been reported orally at meetings and MPS I Advisory Boards, where some of the authors have participated in the past years.

However, the use of ERT in this manner, if performed in a fully engrafted patient transplanted from a non-carrier donor, has no rationale because after many years from transplantation the enzyme should be widely available in virtually all tissues. In addition, it is also not clear why ERT could be of benefit in a successfully transplanted patient from a heterozygous donor. If a real benefit could be clearly demonstrated, this might suggest that HSCT and ERT act in different tissues in different ways and would deserve extensive experimental studies to demonstrate if true and how it happens. At present we can state that there is no evidence to justify ERT years after transplant.

### Multidisciplinary approach

Despite the use of HSCT in MPS I Hurler patients, non-progressive mental retardation, which correlates with age at transplant [8, 9], persistent or progressive dysostosis multiplex, eye and ear diseases, respiratory insufficiency/obstruction, and heart valve damage can cause substantial residual disease burden. Consequently, the management and follow-up of MPS I patients in both pre- and post-transplant phases is best achieved by an integrated multidisciplinary team.

Dysostosis multiplex refers to the skeletal disease associated with MPS I. Odontoid hypoplasia, thoracolumbar kyphosis, genu valgum, hip dysplasia and carpal tunnel syndrome (CTS) are the most frequently reported musculoskeletal manifestations of MPS I-H after HSCT [49]. Importantly, many bone defects have already developed prior to HSCT treatment hence even early treatment with HSCT is unable to prevent dysostosis multiplex [49]. Nonetheless, patients’ age at transplantation may influence skeletal outcome after HSCT with age at time of transplantation shown to significantly influence the age at time of CTS surgery (*P* = 0.007) [50].

To preserve musculoskeletal functions and improve the quality of life of MPS I long-term survivor patients after HSCT, a continuous and periodical control must be accomplished by a standardized follow-up procedure that should be shared amongst different treatment centers. Surgical interventions are necessary to correct bone defects of the hip, knee, and vertebral column. Patients should be monitored and operated on in centers with expertise due to their specific characteristics. Unfortunately, standardization of methods for assessing the severity of dysostosis multiplex and of intervention strategies are lacking [49, 51].

Nonetheless, a number of studies have established some assessment criteria. An accurate digitally scored radiographic assessment was used to validate a correlation between radiographic parameters and clinical progression of hip dysplasia [26]. Lower enzyme activity was correlated with poor development of the craniocervical junction, due to dens hypoplasia, in a retrospective study of MPS I-H patients who underwent HSCT [25].

Patients with MPS I also experience increased anesthesiological risks due to frequent obstruction and/or deformities of upper airways, enlarged tongue and reduced mouth opening with consequent severe problems during intubation. In addition, defective craniocervical junctions may represent a risk of hyperextension of the neck. Lastly, heart valve disease present in the vast majority of individuals also increases the risk. Therefore, when MPS I patients require general anesthesia this should only be performed by anesthesiologists experienced with MPS, operating in qualified centers [2, 52].

A scheme for a standardized follow-up procedure has been proposed in order to define core elements for the regular, long-term assessment of MPS I patients. The recommended minimal schedule of assessments for patients with MPS I includes: medical history, general appearance, anthropometry, vital signs and standard medical evaluation; clinical assessments (neurologic, auditory, ophthalmologic, respiratory, cardiac, musculoskeletal, gastrointestinal); laboratory tests; neuropsychological follow-up with DQ/IQ standardized tests according to age and functional outcome measurements (including assessment questionnaires on functional ability and quality of life) as well as the recommended intervals for follow-up assessments [2]. This guidance may represent an effective tool to coordinate the activity of an MPS I-dedicated multidisciplinary team. However, it must be noted that the different specialists operating in the team should adapt the actual assessment schedule according to the requirements of individual patients.

It is also of paramount importance to standardize data collection, so that comparisons of patient outcome within a center and among different centers is unbiased and meaningful. Moreover, the standardization among different centers of diagnostic and therapeutic procedures, evaluation scores and follow up examinations are obtainable through shared, up-to-date and comprehensive guidelines, which must contemplate the extreme variability of phenotypic evolution of the single patient. This variability must be carefully evaluated by the specialist team and physicians shall consider guidelines merely as a starting point, which may be modified according to the patients’ needs.

## Conclusions

MPS I-H is a complex disorder, mostly due to heterogeneity of *IDUA* mutational profile, which is largely responsible for phenotypical and clinical heterogeneity. Early diagnosis is instrumental in achieving the most appropriate treatment tailored to the different clinical manifestations of MPS I. Newborn screening is a good option for early diagnosis: nowadays regional screening pilot projects are already in place for MPS I measuring IDUA activity using dried blood spots, although reliable markers for early prediction of the phenotype allowing the decision of an appropriate therapeutic intervention have yet to be fully validated. Moreover, verifiable outcome predictors and markers still need to be identified and to be made comparable amongst different reference centers.

Before newborn screening programs are implemented and suitable biomarkers are validated, a set of recommendations can be indicated to achieve the earliest possible diagnosis and the most appropriate intervention.

1. Primary care providers and pediatricians must increase their awareness of MPS I-H, to better recognize the peculiar signs and symptoms and promptly refer the child to a qualified medical center with experience in MPS. The reference centers must include a multidisciplinary integrated team of specialists, who ensure comprehensive management of MPS I-H patients, from diagnosis to treatment and follow-up.
2. For the management of MPS I-H, HSCT is the gold standard therapeutic option in patients younger than 2–2.5 years of age. In any case, when the patient is considered eligible for transplant, HSCT should be performed as early as possible.
3. In the pre-transplant phase, a number of factors influencing the transplant outcome need to be examined, including the age of the child, his/her clinical conditions and the degree of neurological and cognitive development. Moreover, the appropriate selection of graft source and donor type must also be carefully considered before performing HSCT to achieve a stable engraftment and to avoid the development of GVHD.
4. Peri-transplantation ERT has been demonstrated to stabilize the clinical conditions of pre-transplantation patients. Moreover, co-administration of ERT may improve transplantation outcomes due to the positive impact on the residual disease burden persisting in long-term survivors even in the presence of successful HSCT.
5. The residual disease burden following HSCT (non-progressive mental retardation, orthopedic manifestations, and damage to various organs) needs to be longitudinally monitored by examining the clinical outcome, by measuring enzymatic activity and by evaluating donor chimaerism rate. In light of these considerations, a long-term multidisciplinary follow-up is mandatory although its modalities still need to be optimized and standardized among centers.
6. Gene therapy correcting *IDUA* deficiency in MPS I patients is ready to be tested in the preclinical setting. This approach is promising and will possibly become the most effective and conclusive strategy for the cure of this severe disorder.
7. Finally, diagnostic and therapeutic protocols, data collection, data rating scale, and tests performed at follow-up, must be standardized to allow a proper and full comparison of different cases in order to collect the critical mass of information allowing the thorough and complete study of this rare, life-threatening disorder.
